# Micro-autologous Fat Transplantation (MAFT) for Forehead Volumizing and Contouring

**DOI:** 10.1007/s00266-017-0883-2

**Published:** 2017-04-27

**Authors:** C. K. Chou, S. S. Lee, T. Y. Lin, Y. H. Huang, H. Takahashi, C. S. Lai, S. D. Lin, T. M. Lin

**Affiliations:** 1 Yuan’s General Hospital, No.162, Chenggong 1st Rd., Lingya Dist., Kaohsiung City, 802 Taiwan; 2 Department of Plastic Surgery, Kaohsiung Medical University, No.100, Ziyou 1st Rd., Sanmin Dist., Kaohsiung City, 807 Taiwan; 3 Division of Traumatology, Department of Emergency, Kaohsiung Medical University, No.100, Ziyou 1st Rd., Sanmin Dist., Kaohsiung City, 807 Taiwan; 4 Charming Institute of Aesthetic and Regenerative Surgery (CIARS), 2F.-1, No.172, Ziqiang 2nd Rd., Qianjin Dist., Kaohsiung City, 801 Taiwan; 5 Department of Post Baccalaureate Medicine, Kaohsiung Medical University, No.100, Ziyou 1st Rd., Sanmin Dist., Kaohsiung City, 807 Taiwan

**Keywords:** Fat graft, Forehead, Micro-autologous fat transplantation (MAFT)

## Abstract

**Background:**

Frontal fullness in Asians is often considered to indicate one’s public popularity and leadership skills. Numerous materials and techniques have been applied clinically to recontour or volumize the frontal area, with variable results. The micro-autologous fat transplantation (MAFT) technique proposed by Lin et al. (2nd academic congress of Taiwan Cosmetic Association Taipei, Taiwan) in 2007 has demonstrated its feasibility in facial rejuvenation. In the present study, we used an innovative instrument to apply the MAFT technique to frontal augmentation with fat grafting and reported the results.

**Methods:**

MAFT was performed on 178 patients (167 female, 11 male) during a 5-year period starting in January 2010. Fat was harvested by liposuction, processed and refined by centrifugation at 1200×*g* for 3 min. The purified fat was micro-transplanted for frontal contouring with the assistance of an instrument, the MAFT-GUN. The patients were followed up regularly, and photographs were taken for comparison.

**Results:**

On average, the MAFT procedure took 52 min to complete. The average amount of delivered fat was 10.2 mL. The follow-up period was 34 months on average. No complications, including neurovascular injury, skin necrosis, abscess, nodulation, calcification or irregularity, were noted. A patient-rated satisfaction 5-point Likert scale demonstrated that 83.1% of all patients had favorable results (48.3% were satisfied, and 34.8% were very satisfied).

**Conclusion:**

The concept and technique of MAFT has changed fat grafting from an operation with unpredictable clinical results to an easy, reliable and consistent procedure. Furthermore, the use of a precisely controlled instrument enabled surgeons to perform highly accurate micro-fat grafting. In comparison with other strategies for volume restoration, the MAFT procedure demonstrated high patient satisfaction with the long-term results. Therefore, the use of MAFT as an alternative approach to forehead contouring and volumizing was addressed.

**Level of Evidence IV:**

This journal requires that authors assign a level of evidence to each article. For a full description of these Evidence-Based Medicine ratings, please refer to the Table of Contents or the online Instructions to Authors www.springer.com/00266.

**Electronic supplementary material:**

The online version of this article (doi:10.1007/s00266-017-0883-2) contains supplementary material, which is available to authorized users.

## Introduction

Originating from the Latin “frons,” “frontal” means “the forehead or brow.” Anatomically, the top of the forehead is outlined by the hairline, the edge of where hair grows from the scalp. The bottom of the forehead is marked by the supraorbital ridge, the bony feature of the skull above the eyes. Bilateral temporal ridges comprise the lateral boundary of the forehead [1, 2]. In Asian cultures, the coordination and fullness of the shape of the frontal area without either soft tissue deficiency or bony irregularity of the frontal area is believed to indicate prosperity and leadership capabilities. It is also important for the balance and harmony of the face, especially in the lateral and oblique views [3]. A slight convex forehead without wrinkles will add more attractiveness to a person’s face and to the general perceived image [3].

The literature reports the application of some materials to frontal remolding in cases of congenital anomalies or traumatic injuries [4, 5]. A systematic review of the literature has shown that the use of soft tissue fillers for aesthetic contouring/volumizing of the forehead has become popular in the past decade [2, 6]. However, increasing numbers of reports of complications following the use of fillers in frontal injection have been published [7, 8]. Moreover, a high rate of complications, such as allergic reactions (25%), filler material migration (12.5%), injection necrosis and embolism (25%), and foreign body granuloma (37.5%), have been reported [8]. The ideal strategy and material for contouring/volumizing of the forehead have not been reported to date.

Dr. Neuber reported the first fat grafting in 1893 [9]. This procedure has become common due to the ease of harvesting, abundant volume, and lack of rejection reactions. However, the retention rates are unpredictable, and morbidities, such as abscess, cyst formation, nodulation, or neurovascular injury, have been reported [10, 11]. Structural fat grafting has received extensive attention and has demonstrated acceptable clinical outcomes [12]. Sahin et al. presented their lipofilling on the forehead and achieved effective results in reducing forehead wrinkles and correcting the contour [3]. Our group proposed the concept of micro-autologous fat transplantation (MAFT) in 2006 and demonstrated the reliability of this technique in facial rejuvenation [13–19]. In this study, we clinically performed the MAFT technique for volume restoration of the forehead and achieved favorable long-term results.

## Materials and Methods

### Patient Demographics

Between January 2010 and December 2015, 178 consecutive non-smoking patients (170 female, 8 male) received MAFT for frontal contouring. The exclusion criteria included a history of trauma or other comorbidities, operation or filler injection in the frontal area. Regular follow-up was conducted at an outpatient clinic at 1, 3, and 12 months (or longer where possible) after MAFT.

### Pre-operative Planning and Photography

**Fig. 1:**
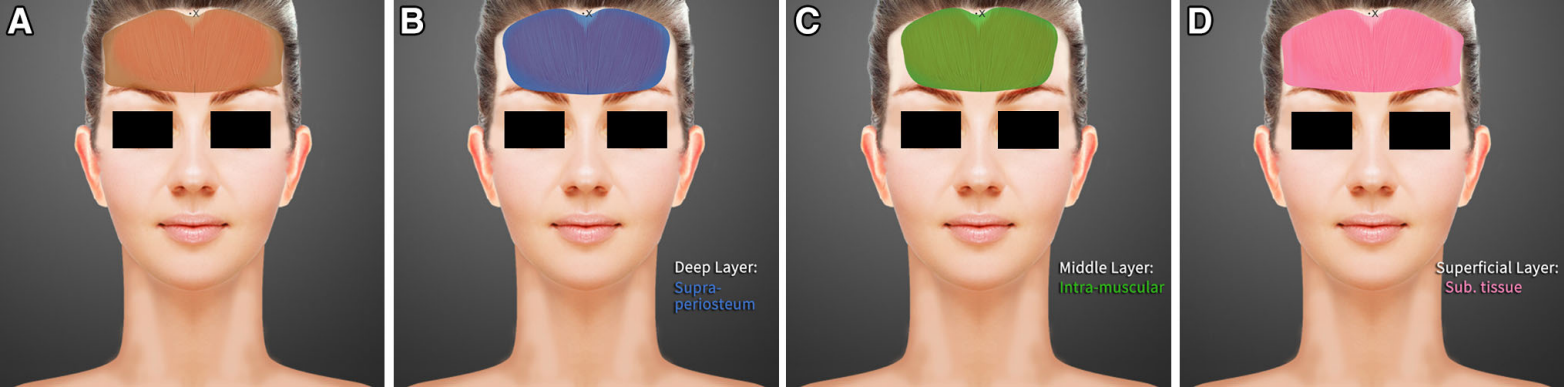
**a** The surgical planning of forehead volumizing and contouring is outlined as a* brown shadow*. The ***X*** point is the insertion site made using a #11 blade. **b** The deep layer is highlighted in *blue* (supra-periosteum, a space between the frontal periosteum and frontalis muscle) to be bounded by the frontal hairline, bilateral temporal ridges and super-orbital ridges. **c** The middle layer in *green* (intra-frontalis muscle) is located between the frontalis muscles of the forehead. **d** The superficial subcutaneous layer is shown in *pink* and is designed to act as a smoothing agent over the forehead boundary

The patients underwent general standard pre-operative procedures and photography after providing signed consent. Seven basic photographs, including the AP view, quarter views (right and left), profile views (right and left), chin-up view (waters view), and chin-down view (helicopter view), were taken from each patient. While standing, the surgical planning of the frontal area was outlined as shown in Fig. 1a.

### Anesthesiology

The patients were under total intra-venous anesthesia during the MAFT procedure. The lipoaspirate was harvested mostly from the lower abdomen (or thigh) area after pre-infiltration with a tumescent solution prepared with a ratio of 10 mL of 2% lidocaine (20 mg/mL): 30 mL of Ringer’s lactate solution: 0.2 mL of epinephrine (1:1000). Appropriate local anesthesia was applied to each recipient at the insertion sites, which normally included the pivot point at the central frontal hairline (point ***X*** in Fig. 1a).

### MAFT Procedure

#### Fat Harvesting

**Fig. 2:**
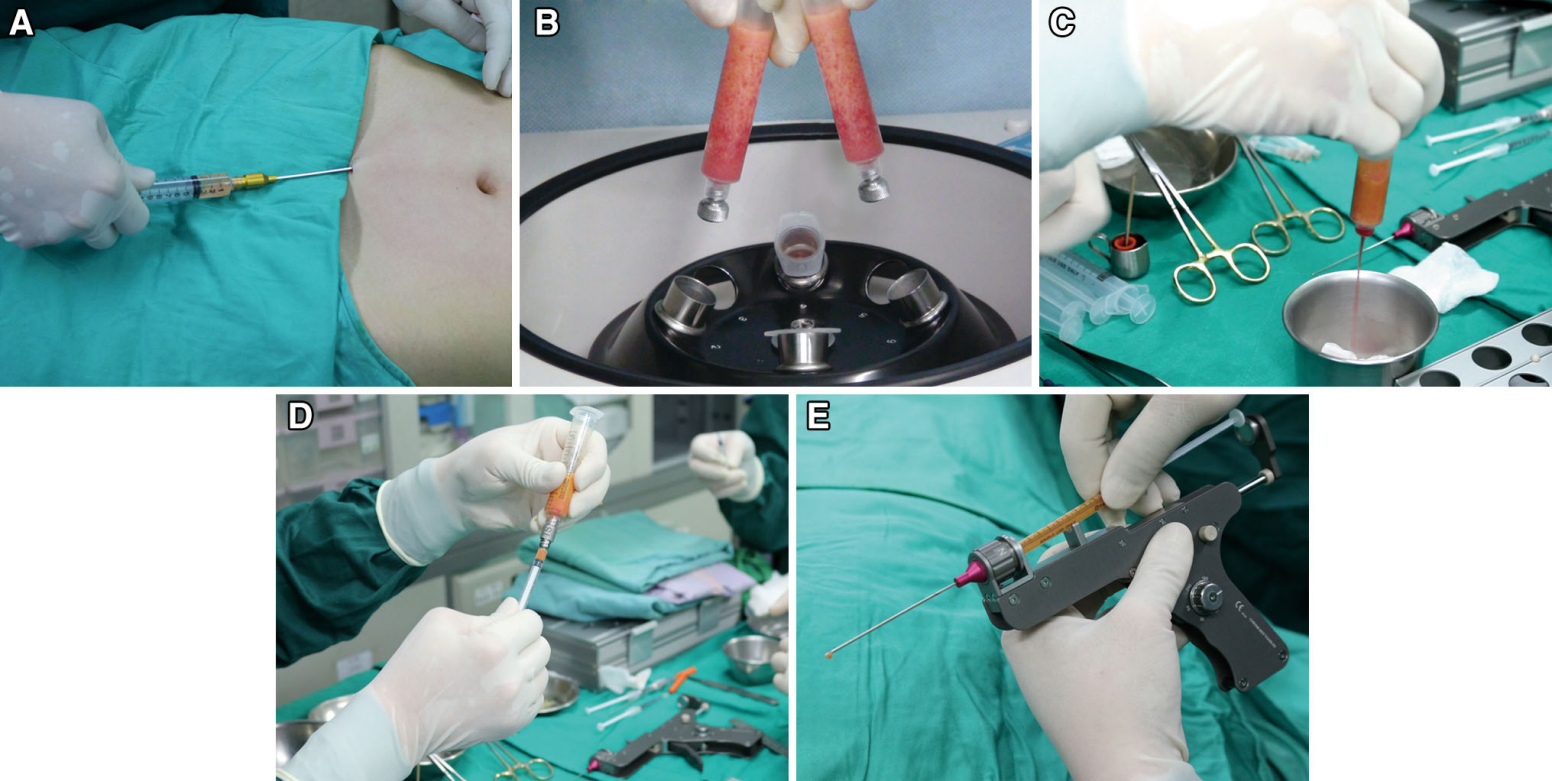
**a** Fat aspiration is performed by back-pulling the plunger of a 10-mL syringe to approximately 2–3 mL to maintain negative pressure. **b** The centrifugation is maintained at 3000 rpm (approximately 1200×*g*) for 3 min to process and purify the lipoaspirate. **c** The *lower part* of the centrifuged lipoaspirate (bloody content) is leaked out, and the oil is wiped off using gauze on the upper part. **d** The purified, condensed fat is transferred to a 1-mL Luer-slip syringe for transplantation. **e** The fat-filled syringe is loaded into the MAFT-GUN for the MAFT procedure. The six-graded dial is set at 120, indicating that the delivered volume of each fat parcel is 1/120 mL (0.0083 mL)

First, a tumescent solution was injected at the donor site; after 10–15 min, the fat was harvested using a blunt tip suction cannula (diameter, 2.5 or 3.0 mm, one side hole). The lipoaspirate volume approximated the amount of injected tumescent solution to ensure a high ratio of purified fat after processing by centrifugation. Low pressure was generally adopted during liposuction to minimize damage to the lipoaspirate. According to Lin et al., the plunger of a 10-mL syringe connected to the suction cannula was pulled back to maintain 2–3 mL of empty space, thereby creating a negative suction pressure of approximately 270-330 mm of mercury (mmHg) (Fig. 2a) [14].

#### Fat Processing and Refinement

The lipoaspirate was poured into a 10 mL syringe screwed on a centrifugation cap to prepare it for centrifugation. In accordance with the “structure fat grafting” technique proposed by Coleman [12], the extracted lipoaspirate was processed and purified by standard centrifugation at 3000 rpm (approximately 1200×g) for 3 min. The lipoaspirate-filled syringes were balanced by placing the syringes in the centrifuge machine in pairs opposite each other (Fig. 2b).

#### Fat Transfer

The centrifuged lipoaspirate was processed and refined by releasing the bottom of the lipoaspirate-filled syringe containing a blood-infused tumescent solution. The oil on top of the syringe was wiped away, leaving only the middle portion containing purified fat to be used for the transplantation. Then, the purified fat was transferred from the 10 mL- to a 1-mL syringe by connecting the two syringes via the syringe transducer (Fig. 2c, d).

#### Fat Transplantation

**Fig. 3:**
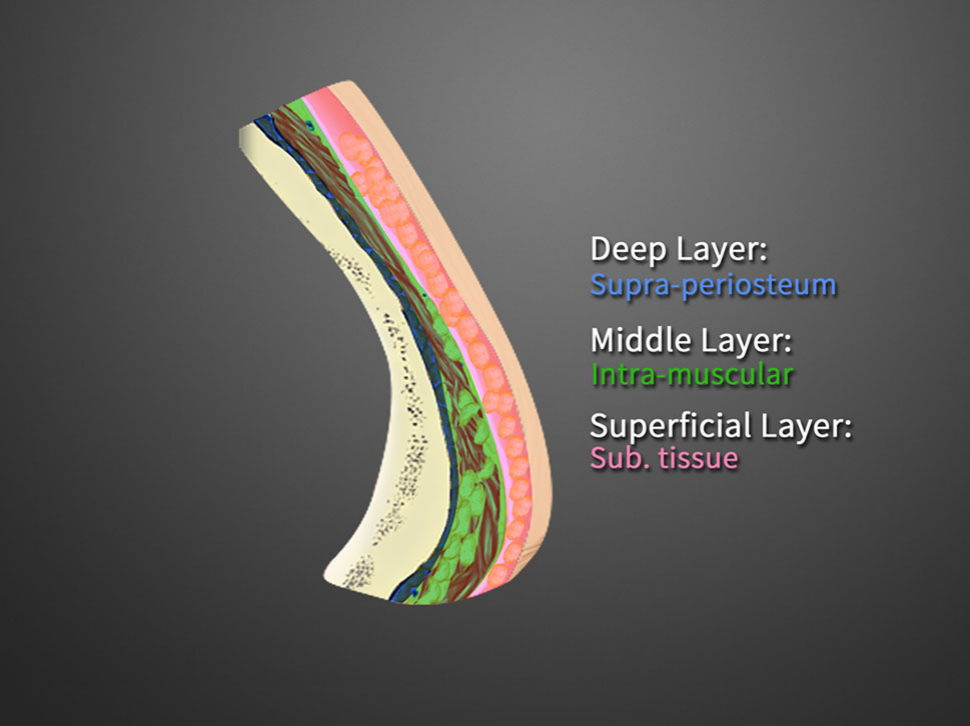
The coronal section of the forehead is shown to illustrate the deeper layer (*blue*), middle layer (*green*) and superficial layer (*pink*) with the transplantation of micro-fat parcels with a size of 1/120 mL (0.0083 mL)

After the purified fat was transferred, the fat-filled syringe was loaded into a MAFT-GUN (Dermato Plastica Beauty Co., Ltd. Kaohsiung, Taiwan) (Fig. 2e). The volume of the fat parcel injected by each trigger was set by adjusting a 6-graded dial to control the total injection aliquot per 1 mL of fat graft. An 18G blunt cannula was employed to inject the fat while withdrawing the MAFT-GUN. Each delivered fat parcel was set at 1/120 mL (each parcel volume 0.0083 mL) and was meticulously transplanted in 3 levels to the frontal area: the deeper layer, on top of the frontal bone; the middle layer, intra-frontalis muscle; and the subcutaneous layer (Figs. 1b–d, 3). The maneuver performed to transplant the fat graft is demonstrated in the “Supplemental Digital Content” (Animation and DVD).

*MAFT Maneuver Technique* (Figs. 1, 3, Animation and DVD)

1. *Fat parcels in deeper layer* (retro-frontalis muscle, on top of periosteum of frontal bone):
  First, make sure the blunt tip of the injection cannula is inserted vertically through a 2–3 mm wound cut using a #11 blade in the center of the frontal hairline. Then, maintain slight pressure on the tip of the cannula and advance into the retro-frontalis space in a fan-shape to the very far end. At this moment, the blunt tip is sliding on top of the periosteum; this maneuver allowed the easy and safe advancement of the full length of the injection cannula to around the eyebrow area (Figs. 1, 3, Animation and DVD). Fat parcels are delivered out of the side hole while the surgeon withdraws the MAFT-GUN. In a fan-shaped manner, numerous parcels (1/120) can be consistently micro-transplanted by triggering the MAFT-GUN. Typically, 5–10 mL (a more or less individual variable) of fat is grafted into this layer.
2. *Fat parcels in middle layer* (intra-frontalis muscle):
  In this layer, once the cannula tip enters the incision at a slight slant (i.e., not vertically), the frontalis muscle layer can be entered. The cannula tip is advanced into the intra-frontalis muscle so the cannula might be more easily advanced between the muscles. Approximately 5–10 mL of the fat parcel can be easily transplanted into this layer.
3. *Fat parcels in superficial layer* (subcutaneous layer, between the dermis and the frontalis muscle):
  While inserting the cannula more superficially upon beginning the insertion, the tip of the cannula advances with resistance into the subcutaneous layer. Meticulously, maintain stable advancement of the injection cannula to prevent penetration of the skin resulting from abrupt exertion. Care should be taken to avoid violent movements and unintentional vascular injury to the supratrochlear or supraorbital artery. As this area is wider compared with the deeper and middle layers, it is not easy to micro-transplant a large number of parcels due to the dense connective tissue (the forehead contains the thickest skin of the face) and the transverse-oriented septa from the skin to the frontalis muscle. Often, the total volume of fat delivered to this superficial layer is only 3–5 mL or less.
4. *Secondary touch*-*up*
  In some patients (22/178, or 12.6% in our study) with relatively thin frontal areas or in patients who wished for more fullness, the placement of additional parcels to achieve a full appearance can be difficult to accomplish in a single session. However, a secondary touch-up MAFT may be performed 4–6 months after the first session to fulfill the patients’ request.

### Post-MAFT Management

Regular postoperative care, including the administration of oral antibiotics and NSAIDs (non-steroid anti-inflammatory drugs), was performed for 3 days when necessary. No massaging was performed following the MAFT procedure. A gentle lymphatic-drain massage was performed 7 days after surgery.

Patient-rated satisfaction was measured anonymously by office staff during the patient’s final visit (6 months after the last MAFT) using a typical Likert scale, with ratings consisting of “very unsatisfied, unsatisfied, neutral, satisfied, very satisfied” (Table 1).

**Table 1:** Patient satisfaction score with micro-autologous fat transplantation (MAFT) for forehead volumizing and contouring (*n* = 178)

	*n* = 178	Very unsatisfied (%)	Unsatisfied (%)	Neutral (%)	Satisfied (%)	Very satisfied (%)
One session	156	0	9(5.8)	21(13.5)	83(53.2)	43(27.5)
Two sessions	22	0	0	0	3(13.6)	19(86.4)
	**178**	0	9(5.1)	21(11.8)	86(48.3)	63(34.8)

Bold value indicates the total number of patients

## Results

**Fig. 4:**
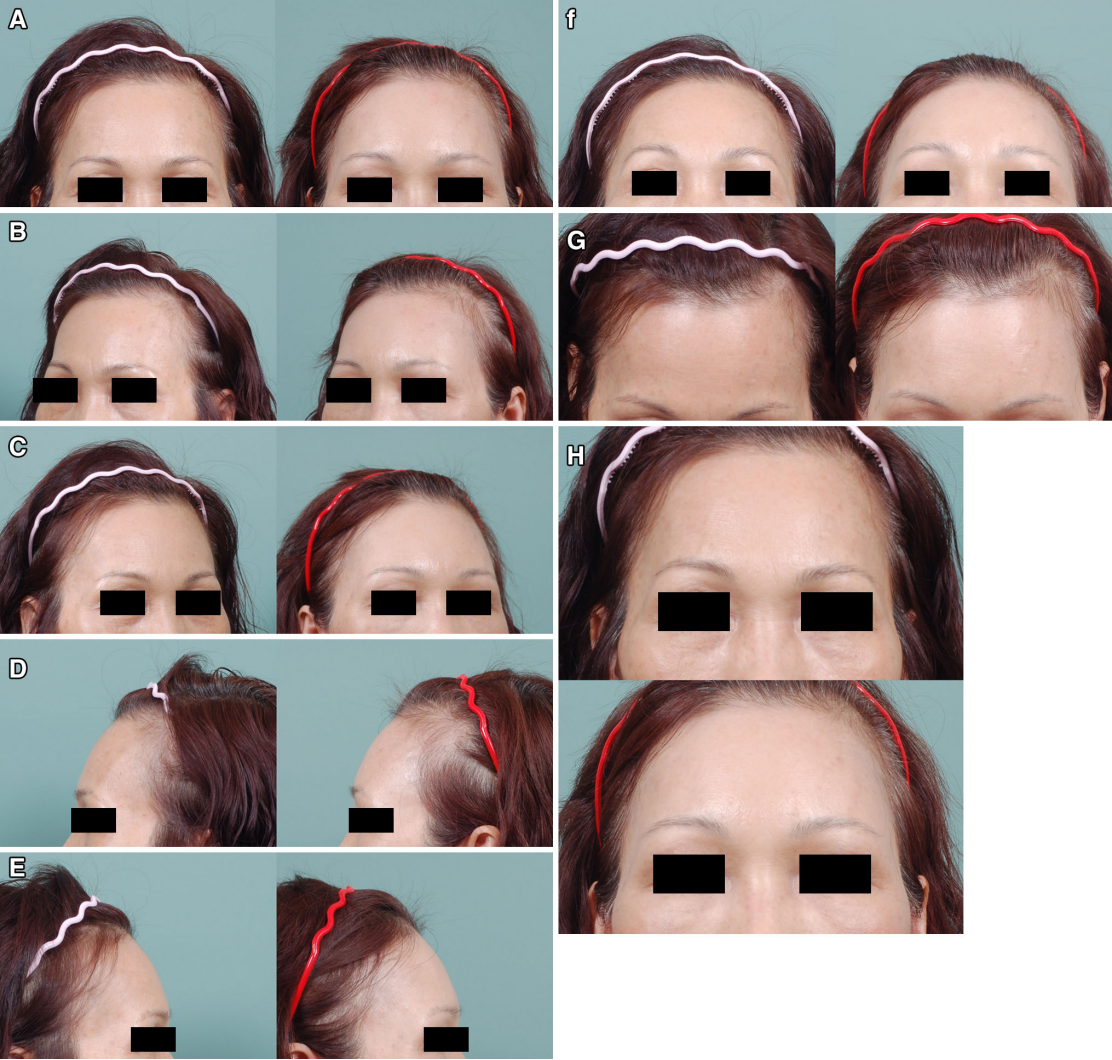
(Case 1) This 54-year-old woman presented for recontouring with fat grafting to increase the youthful appearance of her forehead. MAFT was performed to place a 12-mL fat graft (Pre-op in **a**, **b**, **c**, **d** and **e**, *left*). Six months after a single MAFT session, the volume restoration of the frontal area was maintained with fullness (Post-op in **a**, **b**, **c**, **d** and **e**, *right*). Chin-up, chin-down and close-up views showed the improved contouring (Pre-op in **f**, **g**, **h** and **i** *upper*; Post-op, *lower*)

**Fig. 5:**
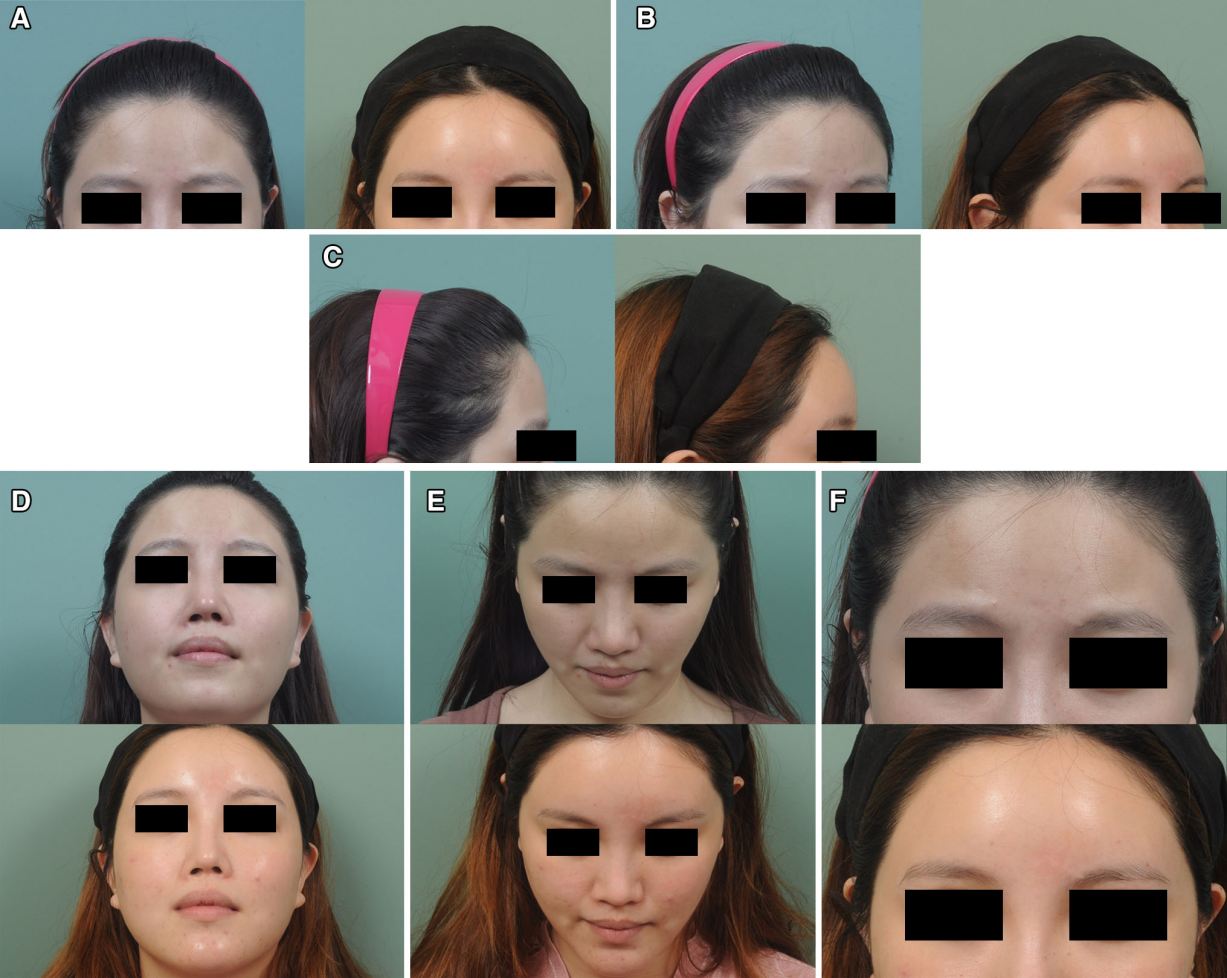
(Case 2) This 25-year-old woman presented for augmentation of her forehead and temporal areas with fat grafting. MAFT was performed on her frontal area to place a 20-mL fat graft (Pre-op in **a**, **b** and **c**, *left*). Two years after a single MAFT session, the volume was maintained on the frontal area (Post-op in **a**, **b** and **c**, *right*). Chin-up, chin-down and close-up views show the improved contouring of the frontal area, which shows a smooth and abundant appearance (Pre-op in **d**, **e** and **f** *upper*; Post-op, *lower*)

**Fig. 6:**
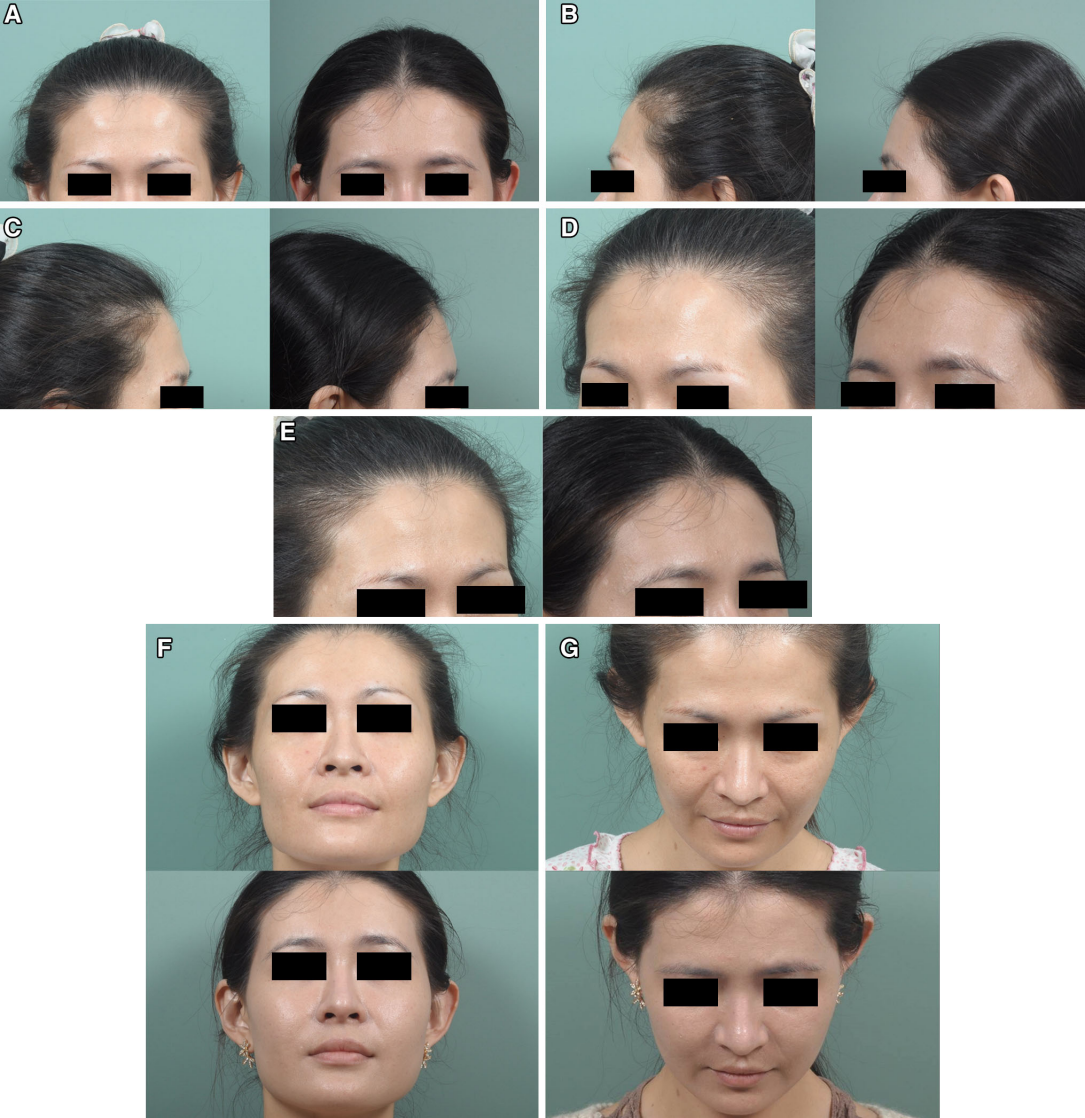
(Case 3) This 33-year-old woman presented for fat grafting to restore her frontal contour. MAFT was performed to place a 10-mL fat graft (Pre-op in **a**, **b** and **c**, *left*). Six months after a single MAFT session, the fullness and volume restoration of the hollowing frontal area were maintained (Post-op in **a**, **b** and **c**, *right*). The improved appearance of frontal unevenness and the rejuvenating effect of the skin are illustrated in a close-up quarter view (Pre-op in **d** and **e**, *left*; Post-op in *right*). Chin-up, chin-down and close-up views showed the recontouring of her uneven frontal area (Pre-op in **f**, **g** and **h**, *upper*; Post-op in *lower*)

The mean age of the 178 patients was 47.7 years (range, 21–72 years). The entire MAFT procedure (from harvesting to transplantation) took an average of 52 min to complete. On average, the fat volume delivered in this study was 10.2 mL. All patients were monitored for an average of 34 months (ranging from 8 to 68 months). No major complications (e.g., infection, skin necrosis, nodulation, fibrosis, calcification, asymmetry or vascular insults) were recorded. Twenty-two patients asked for a second MAFT procedure as a touch-up refinement. The patient-rated satisfaction scores (Table 1) obtained during their final visits showed that 27.5% (43/156) of patients were very satisfied, 53.2% were satisfied (83/156), and 19.6% (21/156) rated their outcome as neutral after one MAFT session. Twenty-two patients (12.6%, 22/178) requested a second session of MAFT (touch-up) for further augmentation and contouring. The satisfaction scores of patients undergoing two sessions showed that 86.4% (19/22) were very satisfied, and 13.6% were satisfied (3/22). Four cases of MAFT for forehead contouring/volumizing are illustrated in Figs. 4, 5 and 6.

## Discussion

Numerous clinical strategies, such as the application of autologous grafts (e.g., bone grafts and fat grafts), synthetic implants and soft tissue fillers, are available for augmenting the frontal area [4–8]. The most common synthetic implant materials, such as silicone prostheses, polyethylene implants, and bone cement, are reliable and achieve acceptable results in selective cases. However, their long-term feasibilities have not been demonstrated. Potential complications, such as infection, deviation, incompatibility and skeletonization, remain a challenge for surgeons [4, 5]. Although fillers such as hyaluronic acid have recently become popular, these materials are not used in all patients due to their expense, the necessity of repeat injections and the possibility of an allergic reaction [7, 8]. Autologous tissues, such as bone grafts, dermal grafts and fat grafts, are preferable due to their biocompatibility and effectiveness in certain cases [4, 5]. However, in fat grafting, dissatisfaction in terms of unpredictable absorption rates, potential morbidities, and a lack of evidence regarding the long-term results remains an unresolved problem [10, 11].

**Fig. 7:**
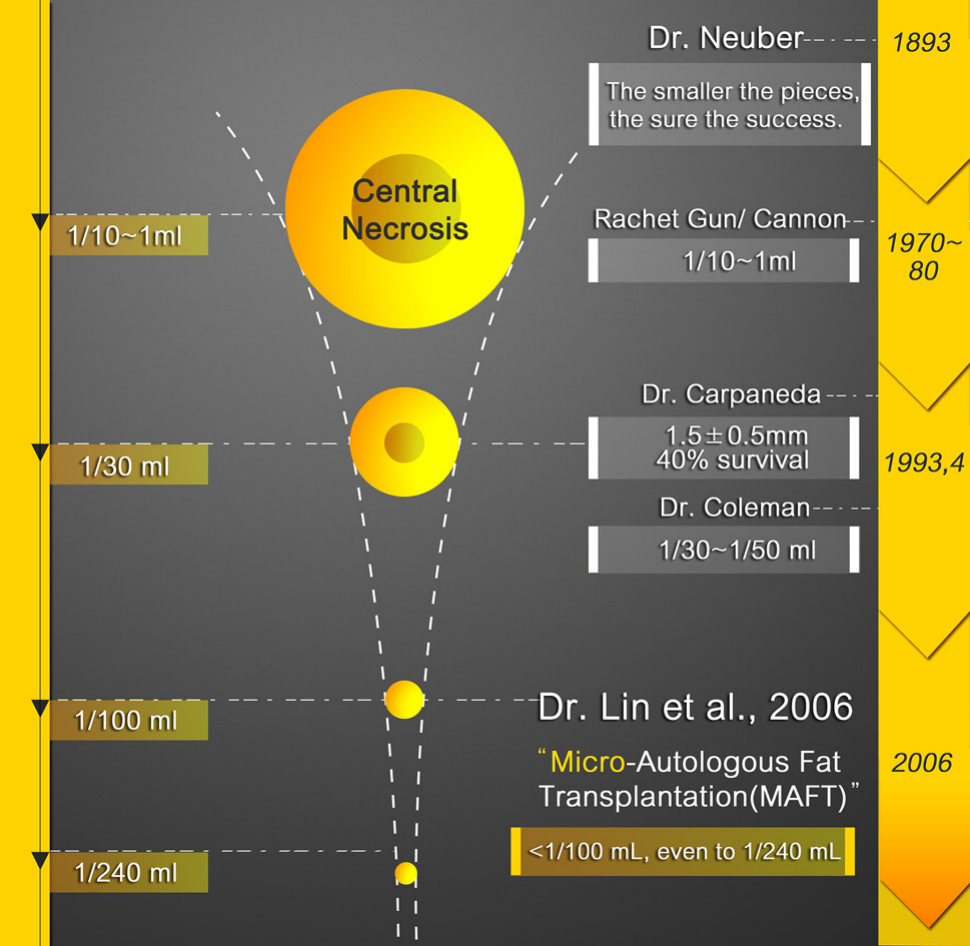
The evolution of the parcel size (volume) was initiated in 1893 by Neuber. The use of the 1/10 mL Rachet Gun per fat parcel (even l mL by larger Rachet Gun) was popular in the 1970–1980s. In 1993, Dr. Coleman proposed that the injection volume for each parcel should be 1/50th–1/30th mL. Moreover, in 1993–1994, Dr. Carpaneda’s theory proposed only a 40% survival rate in the peripheral zone 1.5 ± 0.5 mm of the parcel margin. The conceptualization of MAFT (micro-autologous fat transplantation) was advocated by Dr. Lin et al. in 2006. MAFT emphasizes that each delivered fat parcel is ideally smaller than 1/100 mL (0.01 mL), rendering the real radius of such a parcel to be 1.3 mm

In 1993, Dr. Carpenada emphasized that “only 40% of tissue survived at the area 1.5 ± 0.5 mm to the margin of the fat parcel” [20]. In another word, he emphasized that the central portion of a fat parcel with a radius larger than 2 mm will become necrosed due to poor direct diffusion and impaired plasmatic imbibition in the initial 24–48 h after fat grafting [20]. He also concluded that the percentage of graft viability depends on the thickness and the geometrical shape and is inversely proportional to the graft diameter if grafts with a diameter greater than 3 mm are considered [21]. Therefore, in fat grafting small aliquots are favorable and the ideal radius of the fat parcel is between 1 and 2 mm. By mathematical calculation, the favorable injection aliquot of 1 mL of a fat graft should be between 30 and 240; this was presented as the central dogma of micro-autologous fat transplantation (MAFT) and was advocated by Lin et al. (Table 2; Fig. 7). The concept of MAFT, as proposed by Lin et al. [13], emphasized that each of the delivered parcels should be less than 1/100 mL (<0.01 mL) (i.e., a fat parcel in a spherical shape with a radius of approximately 1.3 mm has a volume of 0.01 mL) to avoid potential fat grafting morbidities (Fig. 7).

**Table 2:** Injection aliquot of 1 mL (1000 mm^3^) fat grafting at 1, 10, 30, 60, 90, 120, 150, 180 and 240 and its corresponding fat parcel radius of 6.20, 2.88, 2.00, 1.58, 1.38, 1.26, 1.17, 1.01 and 1.00 mm, respectively. A spherical volume is calculated with 4/3 × *π* × *r* ^3^ (*r*: the radius of a sphere)

Radius, *γ* (mm)	Volume (mm^3^) 4/3 × *π* × *γ* ^3^	Spherical number (*n*)	1 mL (1000 mm^3^)
1.00	4.2	240	1000
1.01	5.6	180	1000
1.17	6.7	150	1000
1.26	8.3	120	1000
1.38	11.1	90	1000
1.58	16.7	60	1000
2.00	33.3	30	1000
2.88	100	10	1000
6.20	1000	1	1000

When describing the structure of the fat graft, Coleman stated that in specific areas such as the periorbital area (which has thinner skin), each delivered fat parcel should be 1/30–1/50 mL (0.020–0.033 mL) to avoid potential central necrosis and subsequent complications [12]. The patented instrument, the MAFT-GUN, possesses an innovative and precise controlling mechanism that accurately and consistently delivers fat parcels at volumes of 1/60, 1/90, 1/120, 1/150, 1/180 and 1/240 mL. Moreover, the MAFT-GUN provides surgeons with a tool to control the volume of parcels to avoid central necrosis and subsequent complications (Table 2) [13, 14]. The clinical results obtained using MAFT have demonstrated the feasibility of this approach and the importance of controlling the fat parcel size in achieving favorable outcomes [14–19]. Specifically, the accurate and consistent control of the fat parcel volume is critical in avoiding the occasional dislodgement of larger parcels, which results in nodulation and skin irregularity after fat grafting [20–24].

The human forehead is composed of three layers: skin, connective tissue, and muscle. The skin of the forehead is the thickest skin of the face and contains transversely oriented septae extending from the dermis to the frontalis muscle. The vertically oriented frontalis muscle is the main retractor of the upper face and functions primarily to raise the forehead/eyebrows. The fibers of the frontalis muscle originate from the galea aponeurotica on the scalp and insert into the skin of the eyebrows and nose [25]. Therefore, while applying the MAFT technique, we basically divided the soft tissues of the forehead into *3 levels* (*deeper layer*, *middle layer* and *superficial layer*) demarcated by the frontal bone and the frontalis muscle:

- *Deeper layer* (i.e., the retro-frontalis muscle to the frontal bone): the volume of the fat parcels was pre-determined at 1/120 mL, and the 6-grade dial is turned to 120. The fat parcels can be micro-transplanted on top of the periosteum of the frontal bone.
- *Middle layer* (the intra-frontalis muscle): this layer is a relatively well-vascularized layer, providing the grafted fat parcels with a good blood supply.
- *Superficial layer* (subcutaneous layer between the dermis and the frontalis muscle): in this layer, all parcels were transplanted into the subcutaneous tissue.

**Fig. 8:**
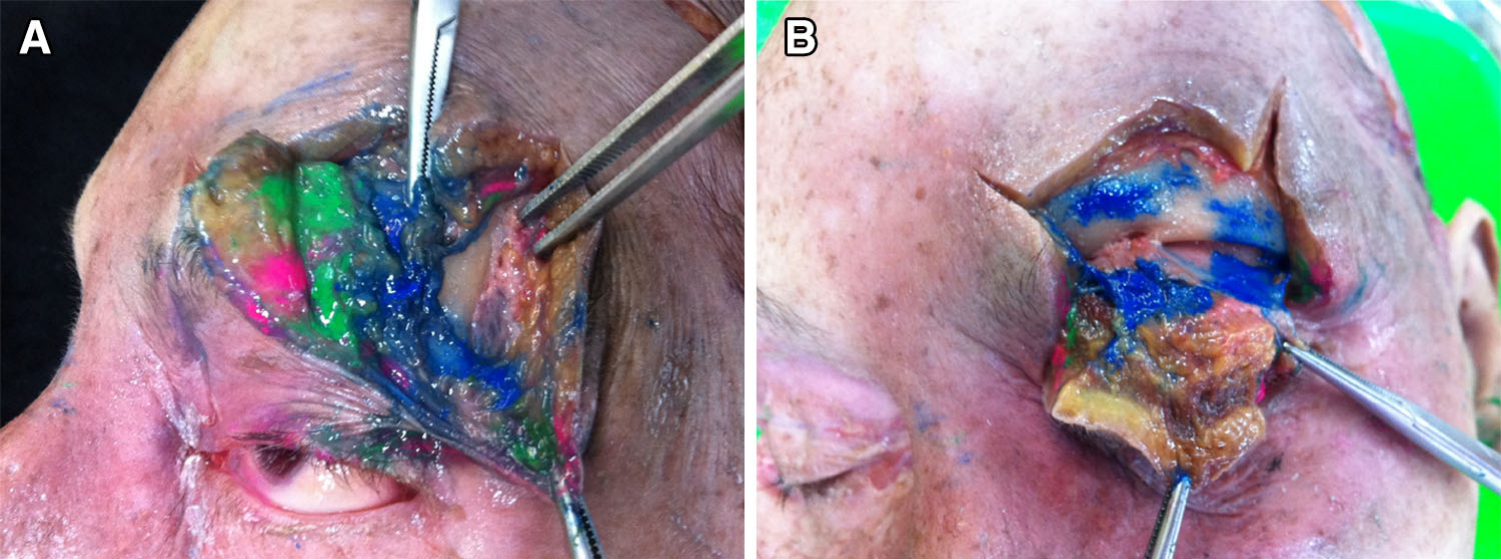
**a** Three dye colors are injected into different layers (*blue* in deep, *green* in middle and *pink* in superficial layer) of the forehead area, as shown in the cadaver dissection. **b** Close-up view demonstrating that the *blue dye* is in the deep supra-periosteum layer, the *green dye* is shown in the middle intra-muscular layer, and the *pink dye* is distributed in the superficial subcutaneous layer

Anatomically, there are 3 levels where the fat parcel should be micro-transplanted, and surgeons should be familiar with these levels. With the use of illustrative dyes (deeper layer, blue; middle layer, green; and superficial layer, pink), surgeons may be familiar with the correct planes for grafting (Fig. 8a, b).

By using the patented instrument, the MAFT-GUN, surgeons can precisely deliver each parcel in the frontal area at 1/120 mL (0.0083 mL) (the actual range of each parcel by the MAFT-GUN can be variably set at 1/60, 1/90, 1/120, 1/150, 1/180 or 1/240 mL) to avoid complications related mostly to central necrosis, which can induce potential morbidities, including abscess, cyst formation, nodulation, severe fibrosis or calcification [10]. Furthermore, vascular compromise, such as intra-vascular injection, a severe side effect of fat grafting [26] is precluded by the MAFT technique due to the following reasons. First, the injection tip cannula is blunt and is therefore safer because it prevents penetration of the supraorbital or supratrochlear arteries during the procedure. Second, in the MAFT procedure, the cannula size is preferably 18G, which corresponds to a diameter of only 1.2 mm. Anatomically, the supratrochlear artery is more superficial and has a larger diameter than the supraorbital artery (1.08 ± 0.19 mm vs. 0.86 ± 0.19 mm) [27]. Theoretically, the 18G injection cannula does not easily achieve intra-luminal penetration. Third, the suggested volume of the fat parcel injected into the frontal area per trigger of the MAFT-GUN is 1/120 mL (0.0083 mL); this volume is very minimal, and the extrusion pressure is relatively low. Therefore, the possibility of blindness or a cerebral vascular accident resulting from an intra-vascular injection with high retrograde flow pressure is almost completely eradicated. Anatomically, the average distance between the midline and the point where the supratrochlear artery crosses the supraorbital rim was 16.4 ± 2.2 mm. While the distance between the midline and the supraorbital artery was 27.2 ± 2.8 mm [27]. When the transplantation is approaching this area, caution should be taken to avoid any unintentional vascular insult. However, meticulous performance without violating the maneuver and appropriate anesthesia to keep the patient calm and stable are also crucial during the MAFT procedure.

Secondary touchups may be considered 4–6 months after the first procedure for those who have undergone one session of MAFT but desire additional volume. The estimated fat retention rate in this study was approximately >50% after one session of MAFT, and the long-term outcome (up to 2 years) was reliable, as anticipated (Fig. 5). However, two sessions of MAFT might be necessary; patients who requested additional fullness or those in whom the frontal soft tissue deficiency was severe were informed pre-operatively of the need for a second session. Due to the increased thickness resulting from fat grafting after the first session, a larger volume of fat might be transplanted in the second session of MAFT to ensure good results.

## Conclusion

Various strategies can be employed to restore forehead contouring in Asian patients. Implants or fillers do not appear to be feasible, appreciated or preferable for all patients. Although not all cases requiring frontal contouring can be solved with one session of fat grafting, MAFT is an alternative strategy of restoring volume and remolding the foreheads of Asian patients.

In conclusion, this study presents the development of a simple and reliable procedure based on the MAFT technique for forehead volume restoration in Asian individuals. Favorable outcomes (80.7%) were obtained for candidates with only one session of MAFT (27.5%, very satisfied and 53.2% satisfied) and 100% (86.4%, very satisfied and 13.6% satisfied) in two sessions of MAFT. The advantages of MAFT in such clinical candidates not only include the recontouring of the forehead but also improving the skin texture with sustainable long-term effectiveness, further confirming that this strategy is a reliable alternative to standard forehead remodeling strategies.

## Electronic Supplementary Material

Below is the link to the electronic supplementary material.

Supplementary material 1 (MP4 6261 kb)

Supplementary material 2 (MP4 154402 kb)
